# Profiles of Wnt pathway gene expression during tooth morphogenesis

**DOI:** 10.3389/fphys.2023.1316635

**Published:** 2024-01-10

**Authors:** Resmi Raju, Jeremie Oliver Piña, Daniela M. Roth, Parna Chattaraj, Fahad K. Kidwai, Fabio R. Faucz, James Iben, Gus Fridell, Ryan K. Dale, Rena N. D’Souza

**Affiliations:** ^1^ Section on Craniofacial Genetic Disorders, Eunice Kennedy Shriver National Institute of Child Health and Human Development (NICHD), National Institutes of Health (NIH), Bethesda, MD, United States; ^2^ Department of Biomedical Engineering, University of Utah, Salt Lake City, UT, United States; ^3^ School of Dentistry, University of Alberta, Edmonton, AB, Canada; ^4^ Molecular Genomics Core, Eunice Kennedy Shriver National Institute of Child Health and Human Development (NICHD), National Institutes of Health (NIH), Bethesda, MD, United States; ^5^ Bioinformatics and Scientific Programming Core, Eunice Kennedy Shriver National Institute of Child Health and Human Development (NICHD), National Institutes of Health (NIH), Bethesda, MD, United States

**Keywords:** tooth morphogenesis, cell differentiation, Wnt signaling modulators, odontoblasts, ameloblasts, dentinogenesis

## Abstract

Mouse and human genetic studies indicate key roles of the *Wnt10a* ligand in odontogenesis. Previous studies have identified effectors and regulators of the Wnt signaling pathway actively expressed during key stages of tooth morphogenesis. However, limitations in multiplexing and spatial resolution hindered a more comprehensive analysis of these signaling molecules. Here, profiling of transcriptomes using fluorescent multiplex *in situ* hybridization and single-cell RNA-sequencing (scRNA-seq) provide robust insight into the synchronized expression patterns of *Wnt10a*, *Dkk1*, and *Sost* simultaneously during tooth development. First, we identified *Wnt10a* transcripts restricted to the epithelium at the stage of tooth bud morphogenesis, contrasting that of *Sost* and *Dkk1* localization to the dental mesenchyme. By embryonic day 15.5 (E15.5), a marked shift of *Wnt10a* expression from dental epithelium to mesenchyme was noted, while *Sost* and *Dkk1* expression remained enriched in the mesenchyme. By postnatal day 0 (P0), co-localization patterns of *Wnt10a*, *Dkk1*, and *Sost* were observed in both terminally differentiating and secreting odontoblasts of molars and incisors. Interestingly, *Wnt10a* exhibited robust expression in fully differentiated ameloblasts at the developing cusp tip of both molars and incisors, an observation not previously noted in prior studies. At P7 and 14, after the mineralization of dentin and enamel, *Wnt10a* expression was limited to odontoblasts. Meanwhile, Wnt modulators showed reduced or absent signals in molars. In contrast, strong signals persisted in ameloblasts (for *Wnt10a*) and odontoblasts (for *Wnt10a*, *Sost*, and *Dkk1*) towards the proximal end of incisors, near the cervical loop. Our scRNA-seq analysis used CellChat to further contextualize Wnt pathway-mediated communication between cells by examining ligand-receptor interactions among different clusters. The co-localization pattern of *Wnt10a*, *Dkk1*, and *Sost* in both terminally differentiating and secreting odontoblasts of molars and incisors potentially signifies the crucial ligand-modulator interaction along the gradient of cytodifferentiation starting from each cusp tip towards the apical region. These data provide cell type-specific insight into the role of Wnt ligands and mediators during epithelial-mesenchymal interactions in odontogenesis.

## Introduction

Wnt signaling provides critical cues in many processes of embryogenesis, including organ development, tissue homeostasis, and wound repair (Tamura and Nemoto, 2016). Several Wnt signaling components, including Wnt ligands, receptors, transducers, transcription factors, and antagonists, are expressed in the dental epithelium and mesenchyme during tooth development in humans and mice (Kornsuthisopon et al., 2022). Following patterning morphogenesis, *Wnt10a* is strongly expressed in pre- and fully-differentiated odontoblasts (Yamashiro et al., 2007), with expression levels maintained in odontoblasts adjacent to Hertwig’s epithelial root sheath (HERS). *Wnt10a* deficiency causes enamel hypoplasia, leading to a flattened crown and taurodontic roots in mice and humans (Yang et al., 2015).

Several extracellular Wnt antagonists (Malinauskas and Jones, 2014) expressed in the tooth organ either bind directly to or compete with Wnt ligands for binding to the co-receptors, Lrp5 and Lrp6. Dickkopf (Dkk) and Sclerostin (Sost) proteins are secreted Wnt antagonists that block the binding of Wnt ligands to Lrp5 and Lrp6 receptors. Inhibition of epithelial Wnt/β-catenin through *Dkk1* overexpression arrests tooth morphogenesis at early or late bud stage (Han et al., 2011). A previous study reported that *Sost* expression was restricted to odontoblasts at the dentin matrix-secreting stages (Naka and Yokose, 2011). *Sost* overexpression in human pulp-derived odontoblast-like cells inhibited odontogenic differentiation (Liao et al., 2019). Nevertheless, mice lacking the *Sost* gene exhibit alterations only in periodontal bone and cementum, with no other abnormalities observed in tooth structure (Kuchler et al., 2014).

Wnt agonist therapies have been studied to stimulate Wnt signaling for the propagation of dentin-pulp regeneration (Neves et al., 2017; Ali et al., 2019; Birjandi et al., 2020). Mice lacking the *Sost* gene show significantly improved pulp healing following injury (Collignon et al., 2017). Inhibition of DKK1 and SOST using a bispecific antibody has shown superior bone repair activity to that of monotherapy (Florio et al., 2016). Modulating components of the Wnt signaling pathway holds great promise in the field of tooth regeneration. Therefore, it is imperative to gain a comprehensive understanding of the spatiotemporal expression patterns during odontogenesis in embryonic and early postnatal developmental stages. The primary aim of this study is to conduct a robust spatiotemporal profiling of Wnt mediators, specifically *Sost* and *Dkk1*, along with the Wnt effector ligand, *Wnt10a*, during odontogenesis, utilizing multiplex, high spatial resolution technology. Furthermore, our study aims to explore if the expression pattern of these three genes differs between non-growing molars and continuously self-renewing incisors. Profiling of transcriptomes using fluorescent multiplex *in situ* hybridization and single-cell RNA-sequencing (scRNA-seq) provides a distinctive insight into the synchronized expression pattern of *Wnt10a*, *Dkk1,* and *Sost* during tooth development not previously conducted.

## Materials and methods

### Animals and embryo collection

All animal procedures were approved by the National Institutes of Health, National Institute of Child Health and Human Development Animal Care and Use Committee (ACUC), under Animal Study Protocol #21-031. Mice were housed in a temperature-controlled room under artificial lighting with access to food and water *ad libitum*. Healthy fertile male mice were mated with the same strain C57BL/6J female mice to generate embryos and pups at different developmental stages. Embryonic day 0.5 (E 0.5) was identified as the day on which the presence of a vaginal plug was confirmed. Six embryonic and four postnatal stages, regardless of their sex, at each developmental stage (E12.5, E13.5, E14.5, E15.5, E17.5, E18.5, postnatal day 0 (P0), P1, P7, P14 (n = 3 biological replicates and n = 3 technical replicates in each, Total number of pregnant mice used = 30) were used in this study. Mice were euthanized by an overdose of carbon dioxide followed by cervical dislocation.

### Histology preparation

E12.5 to E18.5 embryos, and P0/P1, P7, P14 (n = 3) mice pups were used for morphological observation using hematoxylin and eosin (HE) staining. Whole embryo/pup heads were fixed in 10% neutral buffered formalin (NBF) (Azer Scientific, Cat No. NAF-4-G) for 24 h and dehydrated and processed, paraffin-embedded, and serially sectioned (4.5 μm) in the coronal (molar) and sagittal (incisor) plane. Stages at P0 or later were decalcified using EDTA (0.5M, pH 8.0, Quality Biological, Cat No. 351-027-101). The sections were deparaffinized, rehydrated, stained with H&E, and imaged on an AxioScan.Z1 slide scanner (Zeiss).

### Fluorescent multiplex mRNA *in situ* hybridization (RNAscope)

E12.5 to E18.5 embryos, and P0/P1, P7, P14 mice (n = 3) were used for RNAscope analysis. RNAscope Probe-Mm-*Wnt10a*, *Sost*, *Dkk1* (Advanced Cell Diagnostics; ACD, 401061, 410031, 402521) were designed. RNAscope^®^ Multiplex Fluorescent Detection Kit v2 (ACD, 323110) was used to explore the expression pattern of *Wnt10a*, *Sost,* and *Dkk1* following the manufacturer’s protocol. RNAscope slides were imaged on an AxioScan.Z1 slide scanner (Zeiss) with Plan-apochromat 40x/0.95 objective in four fluorescent channels (DAPI, Cy3, Cy5, Cy7).

### Tooth organ dissection, single cell dissociation

E15.5 embryos and P0/P1 mice pups, n = 1 (collected and pooled 6 tooth organs in 1 sample) were used for single-cell dissociation. Mice embryos/pups were dissected out and transferred into ice-cold phosphate-buffered saline (PBS) in a 10 cm petri dish. Tooth organs were carefully dissected (maxillary and mandibular molars) of each respective stage and were collected in a 1.5 mL tube with 1X PBS with 0.04% bovine serum albumin (BSA) on ice. Samples were collected from mice embryos/pups in each respective age group. Harvested tooth organs were cut into small pieces in ice-cold 1X PBS with 0.04% BSA on ice. For isolating the single cells, tissue pellets were then incubated in 1 mL of TrypLE express enzyme (Thermo Fisher Scientific, Waltham, MA) for 30 min at 37 °C. Cells were collected by passing the suspension through a cell strainer, followed by centrifugation.

### Single-cell RNA-sequencing (scRNA-seq)

Libraries from isolated cells were prepared using the Chromium Next Single Cell 3’ Reagent Kit v3.1 (Dual Index −10x Genomics—protocol CG000315 Rev C), aiming for a total of 8,000 cells per sample. In brief, cell suspensions were loaded onto the Chromium Controller microfluidic device to be partitioned into bar code-containing Gel Bead-In-Emulsions (GEMs). Samples were incubated, and poly-adenylated mRNAs were reverse-transcribed into cDNAs containing a unique molecular identifier (UMI) and a cell barcode unique to each GEM. The GEMs were broken using a recovery agent to produce a bulk pool of barcoded molecules. Additional amplification of the cDNA was performed before proceeding to fragmentation, end repair, A-tailing, and ligation. A sample dual index PCR was performed in order to add the P5, i5, i7, and P7 sequences to the final scRNA-seq library. Finally, samples were pooled, and the library was sequenced using the Illumina NovaSeq 6,000 with the SP reagent Kit v.1.5, resulting in 28 × 90 bp paired-end reads.

### Bioinformatics analysis

Cell Ranger (10X Genomics) was used to process reads into a barcode x count matrix by aligning to the mm10 mouse assembly and using the refdata-gex-mm10-2020-A annotation provided by 10X Genomics. This was performed for each of the samples (P1 mandibular molar, P1 maxillary molar, E15.5 mandibular molar, E15.5 maxillary molar). In total, ∼110,000 cells across all samples, ranging from 7,379 to 20,076 per sample, were obtained from Cell Ranger using its default filtering. We further used the scDblFinder package (McGinnis et al., 2019) with default parameters to identify and remove doublets from analysis in each of the samples. Cells were further filtered based on the percentage of counts associated with mitochondrial genes and the number of detected genes per cell, and we removed cells >3 median absolute deviations (MADs) from the median of the total population using the scuttle package (McCarthy et al., 2017). This resulted in 32,288 cells total (ranging from 1,545 to 6,737 cells per sample). Data normalization, scaling, dimensionality reduction, highly variable feature detection, population sub-setting, data integration, and marker detection were all performed with the Seurat v4 (Hao 2021) package based on the standard workflow. Specifically, the steps were the following: normalization with SCTransform (Hafemeister and Satija, 2019; Choudhary and Satija, 2022) on each sample independently using the glmGamPoi method; integration of all samples using SelectIntegrationFeatures with 3,000 features and FindIntegrationAnchors with cca reduction and 5 anchor features; clustering on 40 principal components with a resolution of 1.0; and FindMarkers using Wilcoxon test. Cell types were assigned post-analysis to clusters after inspection of the marker genes. Cells identified as immune response, muscle, endothelial cells, neural, and myeloid cells were then removed, and the remaining cells were clustered and annotated using the same methods and parameters as above.

Cluster identities were assigned based on previously published gene markers (Chiba et al., 2020; Krivanek et al., 2020; Hermans et al., 2022; Jing et al., 2022) (Supplementary Figure S6).

### CellChat analysis

New Seurat objects were generated, filtered, and clustered using the PopsicleR package for the E15.5 and P1 maxillary molar datasets (Grandi et al., 2022). We followed the standard workflow, filtering out cells with less than 200 features and more than 6,500 (E15.5) or 7,500 (P1), >20% (E15.5) or 25% (P1) mitochondrial, and >50% (E15.5) or 45% (P1) ribosomal genes. Doublets were removed at a threshold of 0.35 (E15.5) or 0.38 (P1). Normalization was performed using default values, followed by regression based on cell cycle genes. We then clustered the dataset at 0.6 resolution and annotated clusters as described above.

Individual CellChat objects were generated from the resultant Seurat objects and analyzed following the single dataset analysis tutorial for each (Jin et al., 2021). Querying the WNT signaling pathway network, we generated several plots to visualize predicted interactions within and between annotated clusters. For both E15.5 and P1 maxillary molar datasets, we generated circle plots and chord diagrams to visualize cellular communication and scatter plots to identify signaling roles for cells using network centrality analysis, focused on the WNT signaling pathway network only.

## Results

### *Wnt10a* is expressed in dental epithelium while Wnt modulators, *Dkk1* and *Sost,* are expressed in the dental mesenchyme during the early stages of tooth development

To confirm the morphological features of the molar and incisor tooth organ at each developmental time point, we performed H&E staining of Formalin-Fixed Paraffin-Embedded (FFPE) embryo head specimens. During the initiation of tooth development [E12.5], a localized thickening of the oral epithelium is visualized in the presumptive molariform field (Supplementary Figure S1). To evaluate the spatiotemporal mRNA expression of *Wnt10a*, *Sost,* and *Dkk1* in the molar and incisor tooth organ we performed RNAscope at each developmental time point using FFPE tissue. *Wnt10a* transcripts were detected in the oral and presumptive dental epithelium but were absent in the mesenchyme of maxillary and mandibular molars (Supplementary Figure S1). *Dkk1* was expressed in the mesenchyme of molars, and *Sost* expression was minimal or absent (Supplementary Figure S1). Similar expression patterns were observed in the incisors (Supplementary Figure S2).

**FIGURE 1:**
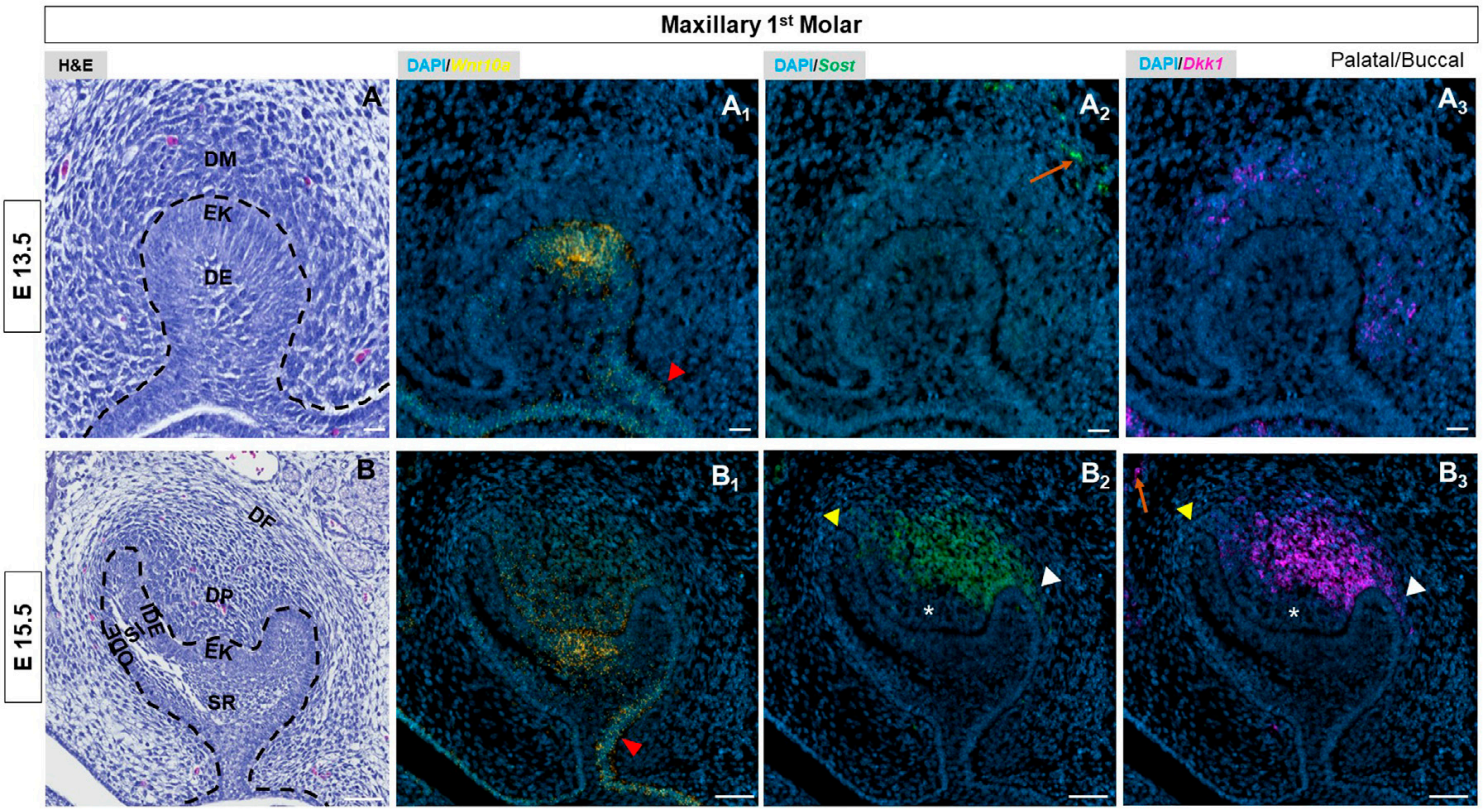
**(A, B)** E13.5 & E15.5 maxillary first molar tooth organs of wild type mice were analyzed using hematoxylin and eosin (HE) staining. RNAscope *in-situ* hybridization of *Wnt10a*, *Sost* and *Dkk1* with positive staining in **(A**_**1**_ **, B**_**1**_ **)** yellow dots **(A**_**2**_ **, B**_**2**_ **)** green dots and **(A**_**3**_ **, B**_**3**_ **)** violet dots respectively, on coronal sections of the maxillary first molar tooth organ from wild-type mice at E13.5 & E15.5. The red arrowhead indicates stronger expression of *Wnt10a* towards the buccal side of the dental epithelium. Yellow arrowhead shows weaker signals of *Sost* and *Dkk1* towards *the* longer palatal cervical loop. White arrowhead shows strong signals of *Sost* and *Dkk1* towards a shorter buccal cervical loop. The absence of *Sost* and *Dkk1* from the coronal papilla area adjacent to the EK is indicated by a white asterisk. Enriched expression of *Sost* and *Dkk1* along the future alveolar bone regions around the tooth organ is marked by an orange arrow. Scale bar, **(A, A**_**1-3**_ **)** 20 µm **(B, B**_**1-3**_ **)** 50 µm. DE-Dental epithelium, DM-Dental mesenchyme, EK-Enamel knot, OD-Odontoblast, AM-Ameloblast, SR-Stellate reticulum, SI-Stellate Intermedium, IDE-Inner dental epithelium, ODE- Outer dental epithelium, DP-Dental papilla, DF-Dental follicle.

By E13.5, the epithelial bud had developed by invaginating into the condensed mesenchyme. The enamel knot (EK) started to appear in first molars at this stage (Figure 1A, Supplementary Figure S3A). *Wnt10a* transcripts were detected throughout the molar bud epithelium with the strongest expression in the EK (Figure 1A_1_, Supplementary Figure S3B). In the dental epithelium, stronger expression was observed towards the buccal side (red arrowhead in Figure 1A_1_, Supplementary Figure S3B), while *Sost* and *Dkk1* were expressed within the mesenchyme (Figure 1A_2_, A_3_, Supplementary Figure S3C, D). At this stage, we found relatively intense expression of *Wnt10a* along the labial border of the epithelial invagination in incisors (white arrow in Supplementary Figure S2).

At the cap stage of molars, [E14.5] (Supplementary Figure S1) *Wnt10a* was exclusively found in the EK area with a slightly stronger signal along the buccal side (Supplementary Figure S1). Enriched expression of both *Sost* and *Dkk1* was observed in the dental papilla (DP) mesenchyme with a noted absence from the coronal papilla area adjacent to the EK (white asterisk in Supplementary Figure S1). A weaker signal of *Sost* and *Dkk1* was noted surrounding the longer palatal cervical loop (CL) (yellow arrowhead) compared to the shorter buccal CL of the maxillary molar (white arrowhead in Supplementary Figure S1).

### *Wnt10a* expression shifts from the epithelium to mesenchyme

At E15.5, the molars and incisors have started to develop into a bell shape (Figure 1B, Supplementary Figure S2, Supplementary Figure S3E). During this stage, *Wnt10a* expression had begun shifting to surrounding mesenchymal cells adjacent to the EK (Figure 1B_1_, Supplementary Figure S3F). We observed a diffuse expression pattern of *Wnt10a* within the EK region. In addition, *Wnt10a* expression was noted in the palatal/lingual region of dental epithelium along with intense expression in the buccal dental epithelium of molars (red arrowhead in Figure 1B_1_, Supplementary Figure S3F). *Sost* and *Dkk1* transcripts in molars did not change from E14.5. The concurrent expression of *Sost* and *Dkk1* was absent in the coronal papilla adjacent to the EK (white asterisk in Figure 1B_2_, B_3_, Supplementary Figure S3G, H). A weaker expression of *Sost* and *Dkk1* around the longer palatal CL (yellow arrowhead) compared to the shorter buccal side of molars (white arrowhead) was noted (Figure 1B_2_, B_3_, Supplementary Figure S3G, H). In incisors, *Sost* and *Dkk1* were expressed in the mesenchyme closer to the EK region (Supplementary Figure S2). Sagittal sections of molars at E16.5 and E17.5 showed patchy expression patterns of Wnt mediators in the DP mesenchyme, with noted absence in areas with intense *Wnt10a* transcripts (Supplementary Figure S5).

### *Wnt10a* and wnt modulators are co-expressed during odontoblast differentiation and dentinogenesis

**FIGURE 2:**
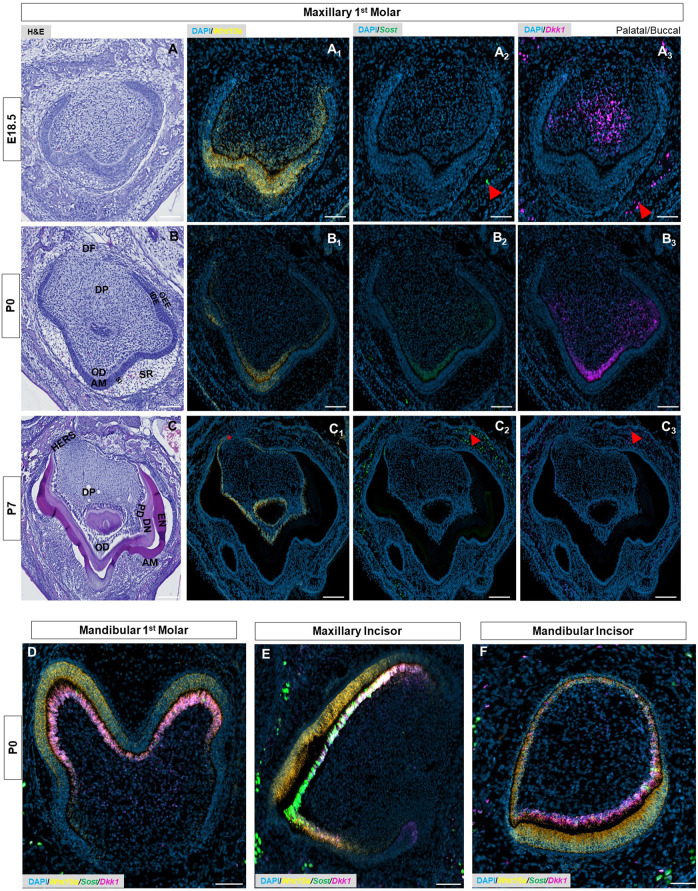
**(A–C)** E18.5, P0 & P7 maxillary first molar tooth organs of wild-type mice were analyzed using hematoxylin and eosin (HE) staining. RNAscope *in-situ* hybridization of *Wnt10a*, *Sost,* and *Dkk1* with positive staining in **(A**_**1**_ **-C**_**1**_ **)** yellow dots **(A**_**2**_ **-C**_**2**_ **)** green dots, and **(A**_**3**_ **-C**_**3**_ **)** violet dots respectively, on coronal sections of the maxillary first molar tooth organ from wild-type mice at E18.5, P0 & P7. **(D–F)** Merged RNAscope *in-situ* hybridization of *Wnt10a*, *Sost,* and *Dkk1* in coronal sections of the mandibular first molar, maxillary and mandibular incisor tooth organ from wild-type mice at P0. The red asterisk shows a strong signal of *Wnt10a* in Elongated HERS. Red arrowhead shows strong signals of *Sost* and *Dkk1* within the osteocytes of developing bone. Scale bar, 100 µm. OD-Odontoblast, AM-Ameloblast, SR-Stellate reticulum, SI-Stellate intermedium, IDE-Inner dental epithelium, ODE- Outer dental epithelium, DP-Dental papilla, DF-Dental follicle, EN-Enamel, DN-Dentin, PD-Pre-dentin, HERS-Hertwig’s epithelial root sheath.

At E17.5 and 18.5 - during the late bell stage (Supplementary Figure S1), (Figure 2A, Supplementary Figure S4A),- *Wnt10a* expression was strongly localized to the inner dental epithelium (IDE) and terminally-differentiating odontoblasts corresponding to the future developing cusps of the maxillary molar (Figure 2A_1_, Supplementary Figure S1). *Sost* and *Dkk1* transcripts in the maxillary molars remained consistent in strength and localization to previous stages (Supplementary Figure S1), (Figure 2A_2_, A_3_). In the mandibular molar, maxillary, and mandibular incisors, *Wnt10a* transcription corresponded to the gradient of terminally differentiating ameloblasts and odontoblasts at the cusp tip regions (Supplementary Figure S2; Supplementary Figure S4). At E17.5, *Sost* and *Dkk1* transcripts were detected strongly throughout the DP of mandibular molars (Supplementary Figure S1). In the incisors at E17.5 and mandibular molars at E18.5, both Wnt mediators were localized to the pre-odontoblasts and terminally-differentiating odontoblasts at the developing cusp tips (Supplementary Figure S2; Supplementary Figure S4C, D).

At P0, secretory odontoblasts were aligned perpendicular to the basement membrane (Figure 2B, Supplementary Figure S4E), and deposited predentin was evident in mandibular molars (black asterisk in Supplementary Figure S4E). Enamel formation was observed at the labial surface of the mandibular incisors (yellow arrowhead in Supplementary Figure S6). *Sost* and *Dkk1* were strongly localized along the secretory odontoblast layer of molars and incisors (Figure 2B_2_, B_3_, Supplementary Figure S4G,H), (Supplementary Figure S6). *Wnt10a* was expressed intensely by the terminally differentiated ameloblasts and odontoblasts along the developing cusp tip of molars and incisors (Figure 2B_1_), (Supplementary Figure S6). Weaker *Wnt10a* expression was noted in the ameloblasts located at the occlusal groove of mandibular molars (red arrow in Supplementary Figure S4F). Likewise, diminished *Wnt10a*, *Sost*, and *Dkk1* expression was noted in odontoblasts located at the occlusal groove region (white arrow in Supplementary Figure S4F-H). This multiplex *in situ* analysis at P0 gives us a clear picture of the co-localization pattern of *Wnt10a*, *Dkk1,* and *Sost* in terminally differentiating and secretary odontoblasts of molars and incisors (Figures 2D–F).

### *Wnt10a* transcripts localized to a single odontoblast layer and wnt modulator gene expression decreases following mineralization of enamel and dentin in the tooth organ

**FIGURE 3:**
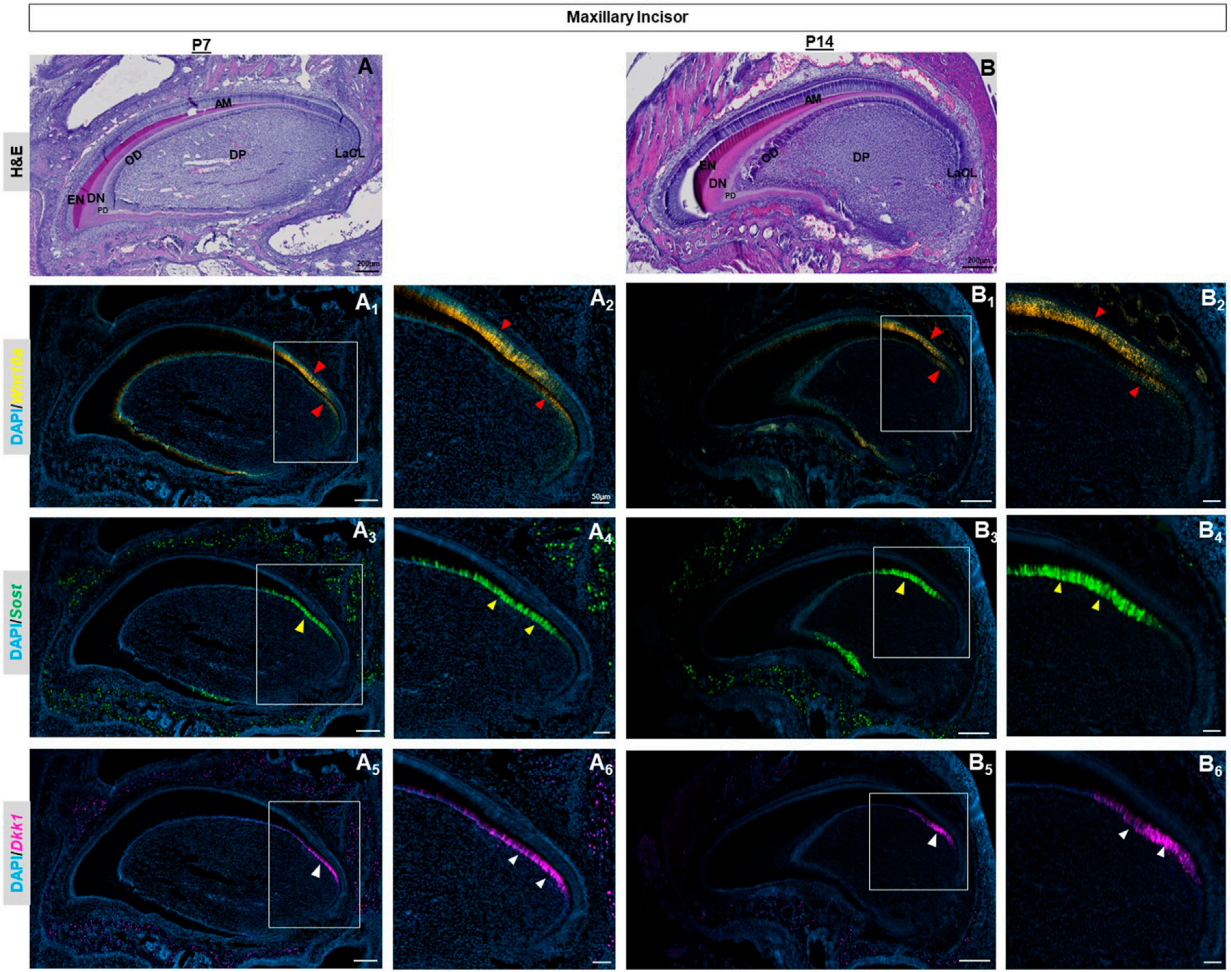
P7 & P14 maxillary incisor tooth organs of wild-type mice were analyzed using hematoxylin and eosin (HE) staining **(A, B)**. RNAscope *in-situ* hybridization of *Wnt10a*, *Sost* and *Dkk1* with positive staining in yellow dots **(A**_**1,**_ **A**_**2**_ **, B**_**1,**_ **B**_**2**_ **)** green dots **(A**_**3,**_ **A**_**4**_ **, B**_**3,**_ **B**_**4**_ **)**, and violet dots **(A**_**5,**_ **A**_**6**_ **, B**_**5,**_ **B**_**6**_ **)** respectively, on sagittal sections of the maxillary incisor tooth organ from wild-type mice at P7 & P14. Intense signals of *Wnt10a* in ameloblasts and odontoblasts towards the proximal end of incisors near the CL are shown by the red arrowhead. The abundance of transcripts of *Sost* and *Dkk1* in proximal odontoblasts near the CL in incisors is marked by yellow and white arrowheads respectively. Scale bar, 200 µm **(A, B, A_1,3,5_, B_1,3,5_)**, 50 µm **(A**_**2,4,6,**_ **B**_**2,4,6**_ **)**, OD-Odontoblast, AM-Ameloblast, DP-Dental papilla, EN-Enamel, DN-Dentin, PD-Pre-dentin, DP-Dental papilla, LaCL-Labial cervical loop.

Advanced mineralization of enamel and dentin occurs in molars and incisors by P7 (Figure 2C, Supplementary Figure S4I), (Figure 3A). *Wnt10a* transcripts were primarily localized to odontoblasts along with a weaker signal in the reduced dental epithelium (RDE) (Figure 2C_1_, Supplementary Figure S4J). Elongated HERS in molars showed a strong signal of *Wnt10a* (red asterisk in Figure 2C_1_, Supplementary Figure S4J). Lower *Sost* expression was exclusively present in some areas of the RDE with a few punctate signals of *Sost* and *Dkk1* observed in odontoblasts near the molar HERS (Figure 2C_2_, C_3_, Supplementary Figure S4K, L). Maxillary incisors showed a strong expression of *Wnt10a* in ameloblasts and odontoblasts along the proximal end of the CL (red arrowhead in Figure 3A_1_, A_2_). Intense signals of *Sost* and *Dkk1* in the odontoblast layer toward the labial CL of maxillary incisors at P7 (yellow and white arrowhead in Figure 3A_3_-A_6_). At P14 we observed weak *Wnt10a* expression in odontoblasts and neither *Sost* nor *Dkk1* was found in molars (Supplementary Figure S7). At P14, intense signals of *Wnt10a* were continuously present in ameloblasts and odontoblasts towards the proximal end of incisors near the CL (red arrowhead in Figure 3B_1_, B_2_). We observed an abundance of transcripts of *Sost* and *Dkk1* in proximal odontoblasts near the CL in incisors (yellow and white arrowhead in Figure 3B_3_, B_6_).

**FIGURE 4:**
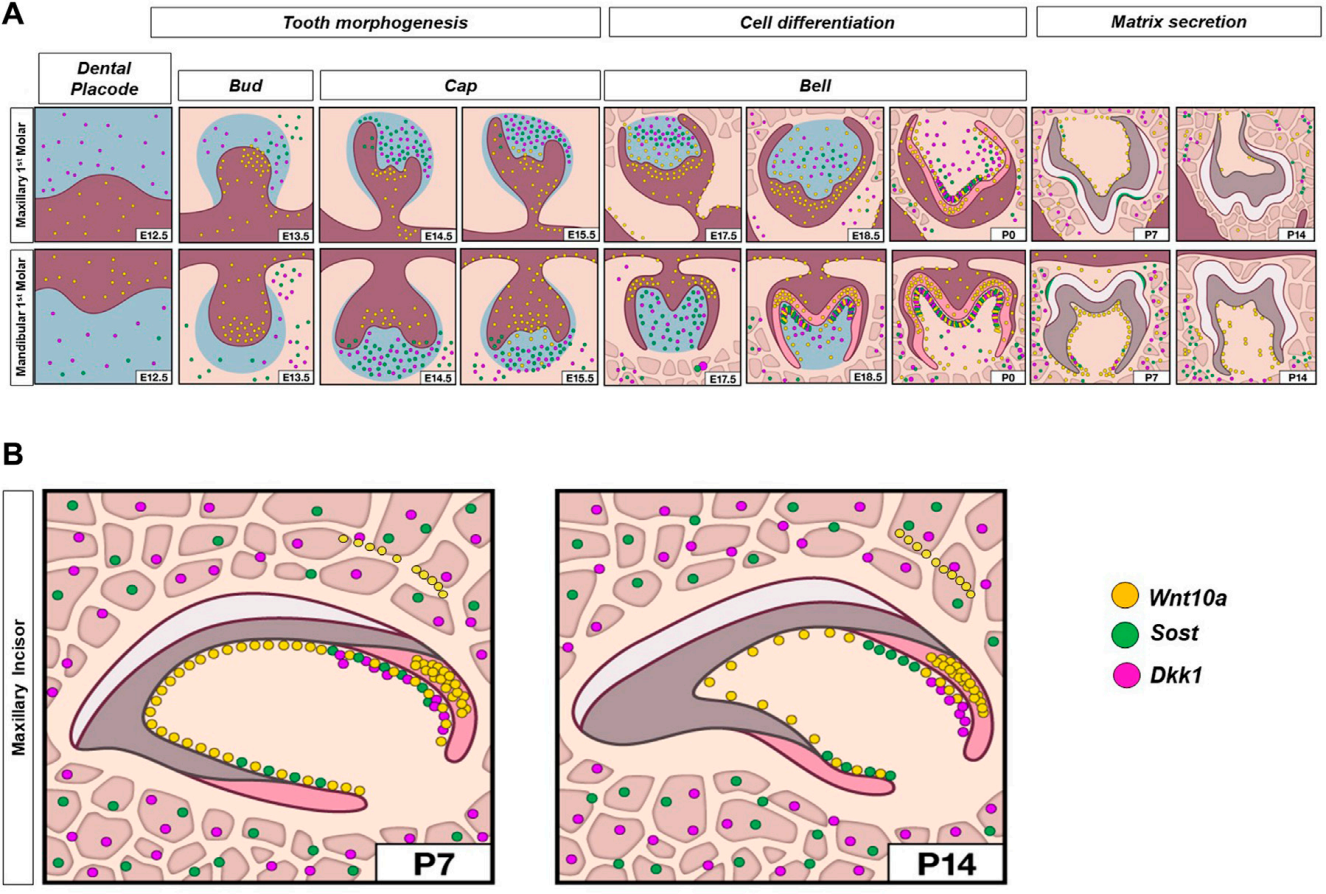
Schematic representation of spatiotemporal expression patterns of *Wnt10a*, *Sost,* and *Dkk1* with positive staining in yellow dots, green dots, and violet dots respectively, on coronal sections of the maxillary and mandibular first molar **(A)**, and sagittal sections of maxillary incisor tooth organ **(B)** of wild-type mice at different developmental stages.

Enriched expression of *Sost* and *Dkk1* was also detected along the future alveolar bone regions around the tooth organ from E12.5 to E15.5 (orange arrow in Figure 1, Supplementary Figure S1-3). During later stages, intense expression of *Sost* and *Dkk1* was found within the osteocytes of developing bone (red arrowhead in Figure 2, Supplementary Figure S4). *Dkk1* and *Sost* expression generally overlapped in expression patterns, and close spatial association was observed throughout development from E14.5 to P0 (Figure 4).

**FIGURE 5:**
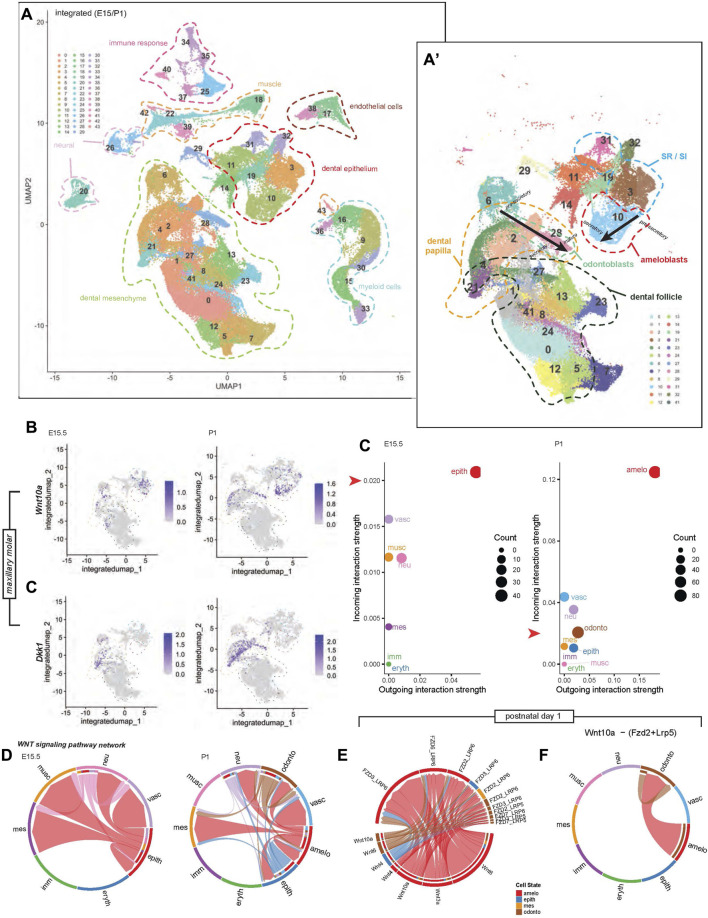
Integrated single-cell transcriptomic profile of murine maxillary first molars at developmental stages E15.5 and P1. **(A)** Uniform manifold approximation and projection (UMAP) highlights 44 unique cell cluster identities. (A′) Sub-clustering of only tooth-specific cell identities enables higher-resolution distinction between key cell types and lineage states; black arrows indicate maturation projection, from pre-secretory, secretory, to mature odontoblasts and ameloblasts. **(B)** Stage-specific feature plots of *Wnt10a* and *Dkk1* expression indicate dynamic spatiotemporal cell cluster enrichment in different cell populations. Low-dimensional depiction of cell clusters in an incoming-outgoing signaling strength at E15.5 and P1 using CellChat. The red arrow along the y-axis of each scatter plot aligns with 0.02 on each plot, highlighting the difference in scale of the incoming interaction strength between plots. **(C)** Similarly, the scale of outgoing interaction strength has a greater range in the P1 plot compared with E15.5. **(D)** Chord diagrams representing the Wnt signaling pathway network between clusters at E15.5 and P1 reconstructed with CellChat. **(E)** Chord diagrams plotting the strength of Wnt signaling between Wnt ligands and receptors at P1. **(F)** Chord diagrams show Wnt10a-(Fzd2+ Lrp6) signaling strength outgoing from ameloblasts and odontoblasts to other cell clusters at P1. Additional colored bars in the inner concentric circles of panels **(D–F)** indicate cell types targeted by outgoing signaling, as these plots are directional. Chord width indicates the strength of flow between the cell types represented in each chord plot. Colors of chord plot links and scatter plot points are coded according to labels along the outside border of the E15.5 and P1 plots in **(D)**, indicating the identity of the outgoing signal. Note: The color corresponding to each cell type is not identical between E15.5 and P1. An example interpretation of panel **(D)** is as follows: At P1, the red link connecting the ameloblast cluster to the odontoblast cluster represents ligands produced by ameloblasts that have known receptors in odontoblasts. The absence of outgoing links from the blue vascular cluster shows that it is not regulating anything but is regulated by other cells (mostly by ameloblasts). However, the ameloblast cluster regulates much more than just vasculature, with the majority of its regulation coming from itself. The abundance of red links in this chord plot overall indicates that the ameloblast cluster is doing the majority of the regulation, and dominates the incoming signal of all types, including to itself.

To profile Wnt signaling genes using single-cell RNA sequencing (scRNA-seq) we first collected the maxillary tooth organ from E15.5 and P1 stages, isolated single cells, and sequenced individual cell transcriptomes. Following this we identified clusters containing dental cell populations in E15.5 and P1 maxillary molars (Figure 5A, A’). Consistent with our RNAscope data, at E15.5, *Wnt10a* was expressed by epithelial-mesenchymal cells and SR/SI. By P1, *Wnt10a* expression was concentrated in odontoblast and ameloblasts. *Dkk1* was absent from epithelial cells at E15.5 and in the ameloblasts at P1 (Figure 5B). Furthermore, we profiled commonly studied Wnt ligands, intermediates, and receptors known to play a role in tooth development. *Wnt4* and *Wnt5b* expression was restricted to the epithelium, while *Wnt11* and mediators *Dkk1*, *Dkk2*, and *Dkk3* were found only in mesenchyme at both stages. In contrast, *Wnt5a* and *Sostdc1* were detected in both epithelial and mesenchymal cells at both stages. Along with *Wnt10a*, *Wnt6* was associated only with epithelium at E15.5 and both epithelium and mesenchyme at P1 (Supplementary Figure S8, 9).

Finally, we used the CellChat tool to predict the contribution of the different cell types to cell–cell Wnt pathway signaling at a lower clustering resolution, differentiating between odontoblast, ameloblast, and other clusters at P1 only. At E15.5, the epithelial cluster is the predominant signal sender and receiver, interacting with the mesenchymal cluster. At P1, the ameloblast-containing cluster had the strongest outgoing and incoming interactions with the odontoblast cluster (Figure 5C). Dental cell-cell interactions at E15.5 were mainly between epithelium-epithelium and epithelium-mesenchyme. At P1, diversification of predicted inter-cluster interactions was reflected by Wnt pathway connections within the ameloblast cluster, between ameloblast-odontoblast, ameloblast-epithelium, and ameloblast-mesenchyme clusters (Figure 5D). At P1, notable Wnt signaling genes associated with ameloblasts and odontoblasts were *Wnt6*, *Wnt10a*, *Wnt3a*, *Wnt4*, and the corresponding receptors, shown in Figure 5E. The strongest predicted Wnt pathway interaction in our dataset was that of *Wnt10a*-Frz2-Lrp5, which was found within odontoblast and between ameloblast-odontoblast clusters (Figure 5F).

## Discussion

The present study provides unique insight into the spatiotemporal relationship of Wnt signaling pathway molecules, which, until now, have not been evaluated across the entire timeline of dental development. This sheds light on the potential interplay between these molecules in facilitating proper tooth morphogenesis. Previous studies have shown that WNT10A phenotypic changes involve microdontia of dentition, defective cusp, and root morphology (Bohring et al., 2009; Mues et al., 2014; Xu et al., 2017). *Dkk1* knockout mice exhibited two significant phenotypes: the absence of anterior head structures and malformed forelimbs (Mukhopadhyay et al., 2001). Elevated expression of *Dkk1* in odontoblasts and dental pulp cells hindered the formation of dentin in mandibular molars and led to pronounced dentin resorption (Han et al., 2011). *Sost*-deficient mice demonstrated an increased width of the cementum and a reduction in the periodontal space width (Kuchler et al., 2014). A better understanding of the dynamic gene expression patterns of Wnt ligands and modulators in dentinogenesis could provide critical information for the development of improved treatments for vital pulp therapy and dentin regeneration.

Our observations differ from prior *in-vivo* studies that reported isolated spatiotemporal expression patterns of *Sost* and *Dkk1* (Fjeld et al., 2005; Naka and Yokose, 2011). It was demonstrated that SOST protein presence was limited to odontoblasts engaged in pre-dentin secretion as well as osteoblasts (Naka and Yokose, 2011). Additionally, at the E13 stage, *Dkk1* transcripts were noted in the epithelial bud of molar tooth germs (Fjeld et al., 2005). Surprisingly, our findings indicate that *Dkk1* and *Sost* transcripts were detected in the adjacent mesenchymal tissue, dental papilla cells, and odontoblasts throughout the developmental stages of both molars and incisors, ranging from E12.5 to P7. Previous studies have shown that the *Sost* gene, acts as an inhibitor of canonical Wnt signaling and is known to play a role in regulating bone mass (Li et al., 2005; Poole et al., 2005; Power et al., 2010). In this study, we have confirmed the presence of *Sost* in dental papilla cells, as well as in secretory odontoblasts located adjacent to the ameloblasts in odontogenesis. Both osteocytes and odontoblasts are responsible for producing the mineralizing matrix and have mechanosensing functions (Lanyon, 1993; Allard et al., 2000). Our research clearly demonstrates that *Sost* also serves as a marker for dental papilla and odontoblast cells during tooth morphogenesis and dentin matrix deposition. Further investigations are necessary to elucidate the mechanisms governing the expression of *Sost* in odontoblasts.

Our data are consistent with earlier studies that implicate *Wnt10a* as potentially involved not only in tooth bud initiation but also in patterning, odontoblast differentiation, and root furcation development (Yamashiro et al., 2007; Zhang et al., 2014; Yang et al., 2015; Xu et al., 2017; Yu et al., 2020; Yoshinaga et al., 2023). Furthermore, our results build upon prior observations by demonstrating that *Wnt10a* mRNA was prominently present not only in terminally differentiated odontoblasts but also in the ameloblasts along the developing cusp tip of both molars and incisors. Robust expression of *Wnt10a* is noted in our study along the gradient cytodifferentiation of ameloblasts at the developing cusp tip of both molars and incisors, suggesting its involvement in the process of crown morphogenesis and amelogenesis. The co-localization pattern of *Wnt10a*, *Dkk1*, and *Sost* in both terminally differentiating and secreting odontoblasts of molars and incisors potentially signifies the crucial interaction between Wnt ligands and mediators originating from each cusp tip and extending towards the apical region. Moreover, there was a decrease in the expression of *Wnt10a* in the ameloblasts, along with reduced expression of *Wnt10a*, *Sost*, and *Dkk1* in the odontoblasts, particularly at the occlusal groove of mandibular molars. This provides further evidence of their crucial involvement in the process of tooth morphogenesis. Following the mineralization of enamel and dentin at P14, there was a noticeable decline in *Wnt10a* signals, with neither *Sost* nor *Dkk1* being detected in molars. It indicates that *Wnt10a* and modulators play a vital role in the deposition of mineralized tissue in tooth development. In addition, prior studies have suggested that *Wnt10a* (Li et al., 2023; Hara et al., 2021) and *Sost* (Collignon et al., 2017) play roles in activating odontoblasts and promoting the differentiation of reparative odontoblasts in response to tooth injury and direct pulp capping. This clearly indicates their role in facilitating mineralized tissue deposition not only in normal dentinogenesis but also in reparative dentinogenesis. We speculate that *Sost* and *Dkk1* become inactive once the mineralized tissue is deposited. However, it is important to note that Wnt ligands and modulators have the potential to be reactivated during the process of reparative dentinogenesis (Li et al., 2023; Hara et al., 2021; Collignon et al., 2017). At P14, high expression of *Wnt10a* transcripts was seen in ameloblasts and odontoblasts along with *Sost* and *Dkk1* in odontoblasts at the proximal end of the growing incisor. These data substantiate the requirement of *Wnt10a*, *Sost*, and *Dkk1* by odontoblasts during the active phases of matrix synthesis, particularly evident at the apical end of continuously erupting incisors.

Our scRNA-seq analysis revealed overlapping or individual expression signatures of Wnt ligands, receptors, mediators, and inhibitors in the dental epithelium and/or mesenchyme, inferring their respective involvement in the differentiation of various cells during odontogenesis. scRNA-seq analysis revealed distinct patterns of expression of *Wnt10a* and *Dkk1* at E15.5 and P1, corroborating and validating our *in-situ* data. This novel dataset now provides a valuable resource for understanding shifts in gene expression profiles between epithelium and mesenchyme, one of the fundamental developmental interactions responsible for the patterning and morphogenesis of tooth organs. Of note, our dataset did not detect sufficient *Sost* expression in the samples collected, which we attribute to relatively low expression that was not detected at the sequencing coverage levels used here. Thus, we could not draw specific conclusions regarding *Sost* from our scRNA-seq. Similarly, the expression of *Wnt10a* and *Dkk1* in scRNA-seq was lower than that observed *in situ*. This may be due to the common drop-out effect where expressed transcripts are not detected because of technical sequencing coverage limitations, or not capturing intact, fully dissociated cells of interest in droplets (Qiu, 2022). Furthermore, the limits of resolution in preparing samples at developmental time points may mean that the E15.5 used for scRNA-seq and E15.5 used for hybridization could still differ from each other by a matter of hours, which may be sufficiently different from each other given the dynamic nature of development.

Several studies have used Wnt agonist therapies to stimulate Wnt signaling for dentin-pulp regeneration (Neves et al., 2017; Ali et al., 2019; Birjandi et al., 2020). Here, the spatiotemporal interrogation of dynamic expression patterns of multiplexed *Wnt10a*, *Sost,* and *Dkk1* during tooth morphogenesis reveals novel insight into the critical modulation of Wnt signaling requisite for proper homeostatic signaling, differentiation, and patterning. Further studies of the molecular mechanisms involving *Wnt10a*, *Sos*t, and *Dkk1* in dentinogenesis and reactionary/reparative dentin formation may pave the way toward the development of multi-target strategies for dentin regeneration.

## Data Availability

The data presented in the study are deposited in the GEO repository (https://www.ncbi.nlm.nih.gov/geo/), accession number GSE247224.
