# Supplementary material for: Profiles of Wnt pathway gene expression during tooth morphogenesis
Source: Front Physiol. 2024 Jan 10;14:1316635. doi: 10.3389/fphys.2023.1316635 (PMC10809389; doi:10.3389/fphys.2023.1316635)
Supplement: Supplementary file 1 [file DataSheet1.docx]

Supplementary Material

**
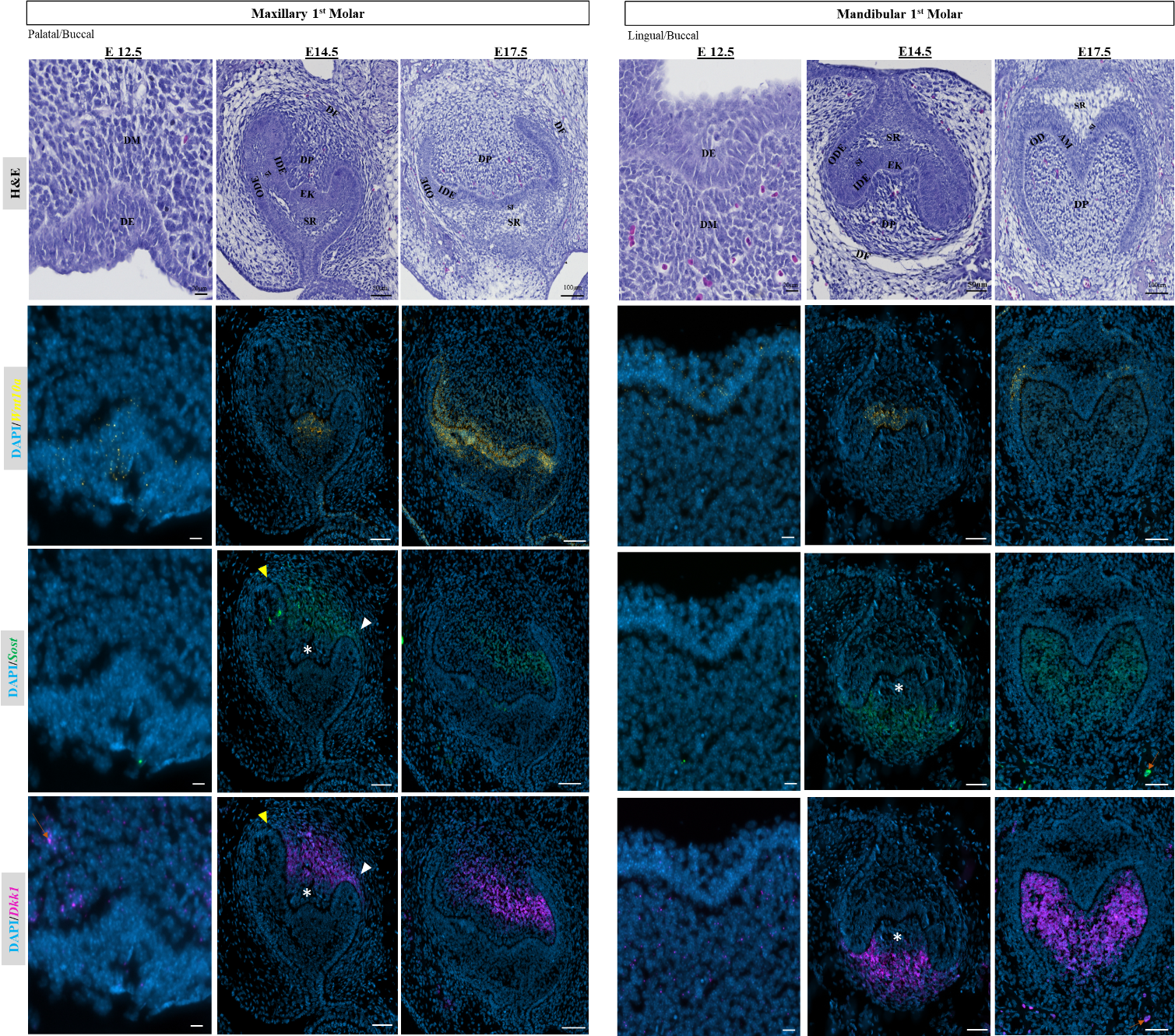
**

**Supplementary Figure 1** Hematoxylin and eosin (HE) staining and RNAscope *in situ* hybridization of *Wnt10a*, *Sost* and *Dkk1*, on coronal sections of the maxillary and mandibular 1^st^ molar tooth organ from wild-type mice at E12.5, E14.5 & E17.5. Yellow arrowhead shows weaker signals of *Sost* and *Dkk1* towards longer palatal cervical loop. White arrowhead shows strong signals of *Sost* and *Dkk1* towards shorter buccal cervical loop. Absence of *Sost* and *Dkk1* from the coronal papilla area adjacent to the EK is indicated by white asterisk. Scale bar, 20µm (all images at E12.5), 50µm (all images at E14.5) and 100µm (all images at E17.5). DE-Dental epithelium, DM-Dental mesenchyme, EK-Enamel knot, OD-Odontoblast, AM-Ameloblast, SR-Stellate reticulum, SI-Stellate Intermedium, IDE-Inner dental epithelium, ODE- Outer dental epithelium, DP-Dental papilla, DF-Dental follicle.

**
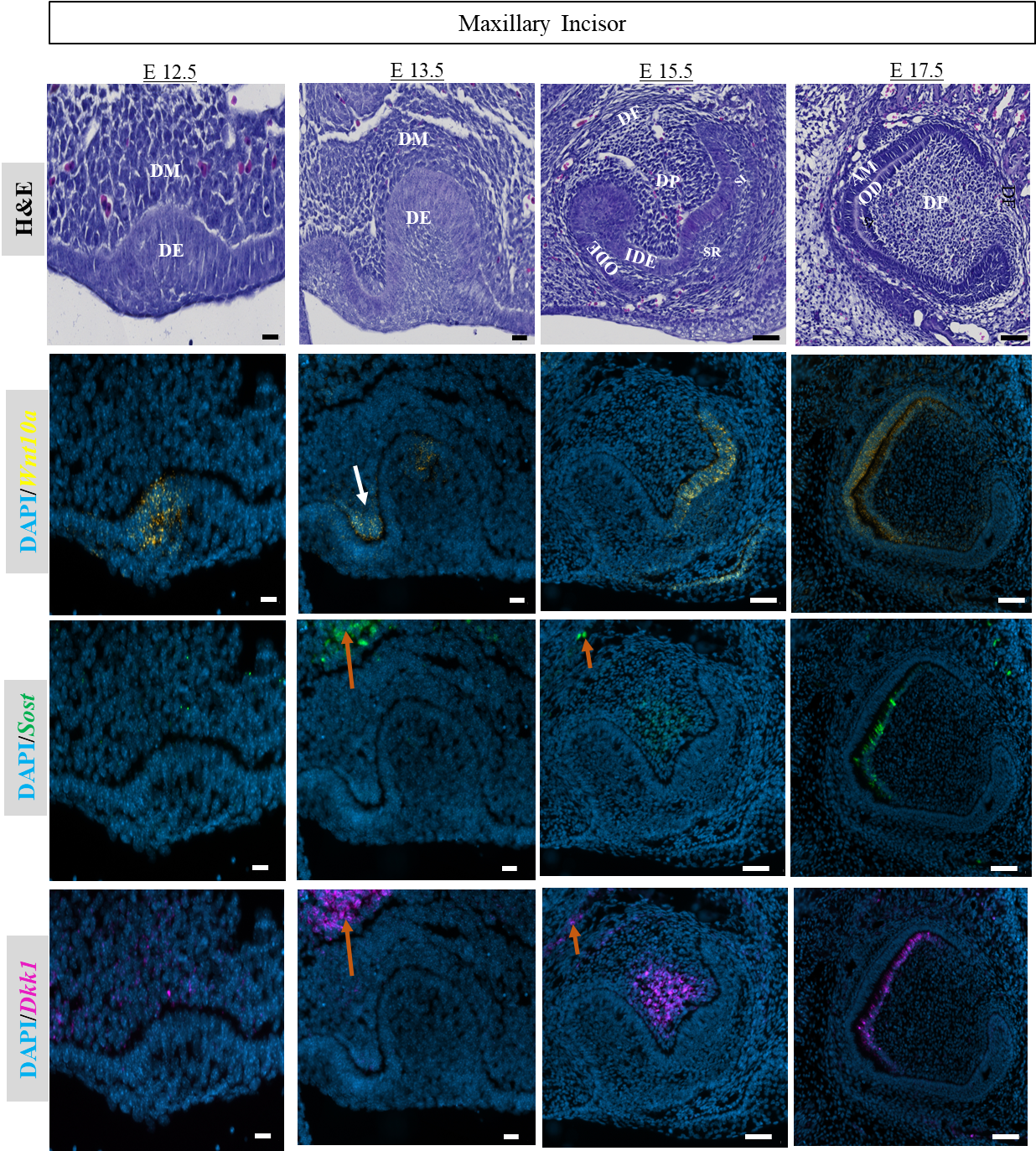
**

**
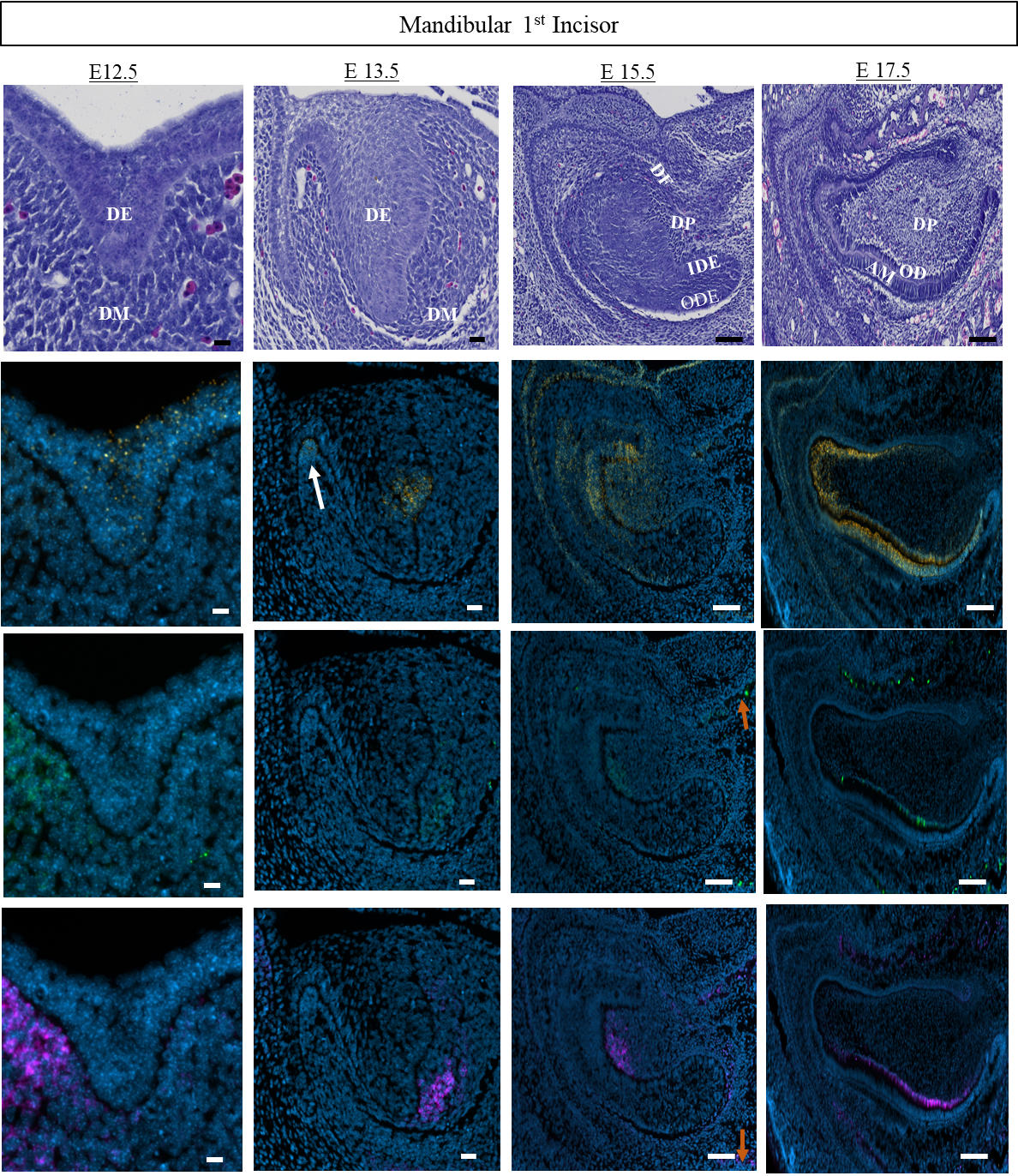
**

**Supplementary Figure 2** Hematoxylin and eosin (HE) staining and RNAscope *in situ* hybridization of *Wnt10a*, *Sost* and *Dkk1* with positive staining in yellow dots, green dots and violet dots respectively, on sagittal sections of the maxillary and mandibular incisor tooth organ at E12.5-17.5. Intense expression of *Wnt10a* along the labial border of the epithelial invagination in incisors is shown by white arrow. Enriched expression of *Sost* and *Dkk1* along the future alveolar bone regions around the tooth organ is marked by orange arrow. Scale bar, 20µm (all images at E12.5 and E13.5), 50µm (all images at E15.5 and E17.5). DE-Dental epithelium, DM-Dental mesenchyme, OD-Odontoblast, AM-Ameloblast, SR-Stellate reticulum, SI-Stellate Intermedium, IDE-Inner dental epithelium, ODE- Outer Dental epithelium, DP-Dental papilla.

**
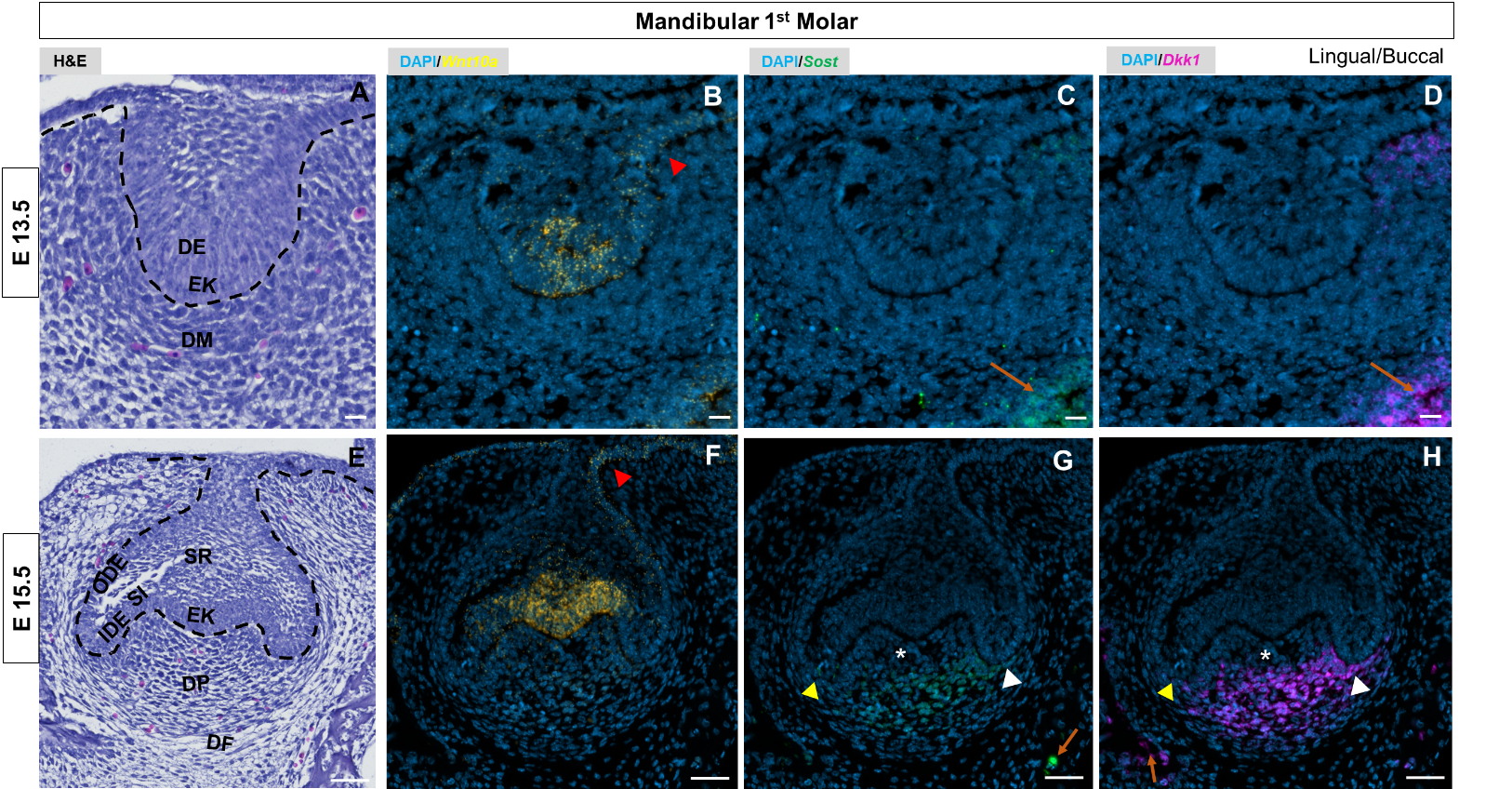
**

**Supplementary Figure 3** Hematoxylin and eosin (HE) staining (**A**, **E**). RNAscope *in situ* hybridization of *Wnt10a*, *Sost* and *Dkk1* with positive staining in yellow dots, green dots and violet dots respectively, on coronal sections of the mandibular molar tooth organ at E13.5 and E15.5. Red arrowhead indicates stronger expression was observed towards the buccal side of dental epithelium. Yellow arrowhead shows weaker signals of *Sost* and *Dkk1* towards longer palatal cervical loop. White arrowhead shows strong signals of *Sost* and *Dkk1* towards shorter buccal cervical loop. Absence of *Sost* and *Dkk1* from the coronal papilla area adjacent to the EK is indicated by white asterisk. Enriched expression of *Sost* and *Dkk1* along the future alveolar bone regions around the tooth organ is marked by an orange arrow. Scale bar, 20µm (A-D) 50µm (E-H). DE-Dental epithelium, DM-Dental mesenchyme, SR-Stellate reticulum, SI-Stellate Intermedium, IDE-Inner dental epithelium, ODE- Outer Dental epithelium, DP-Dental papilla, DF-Dental follicle.

**
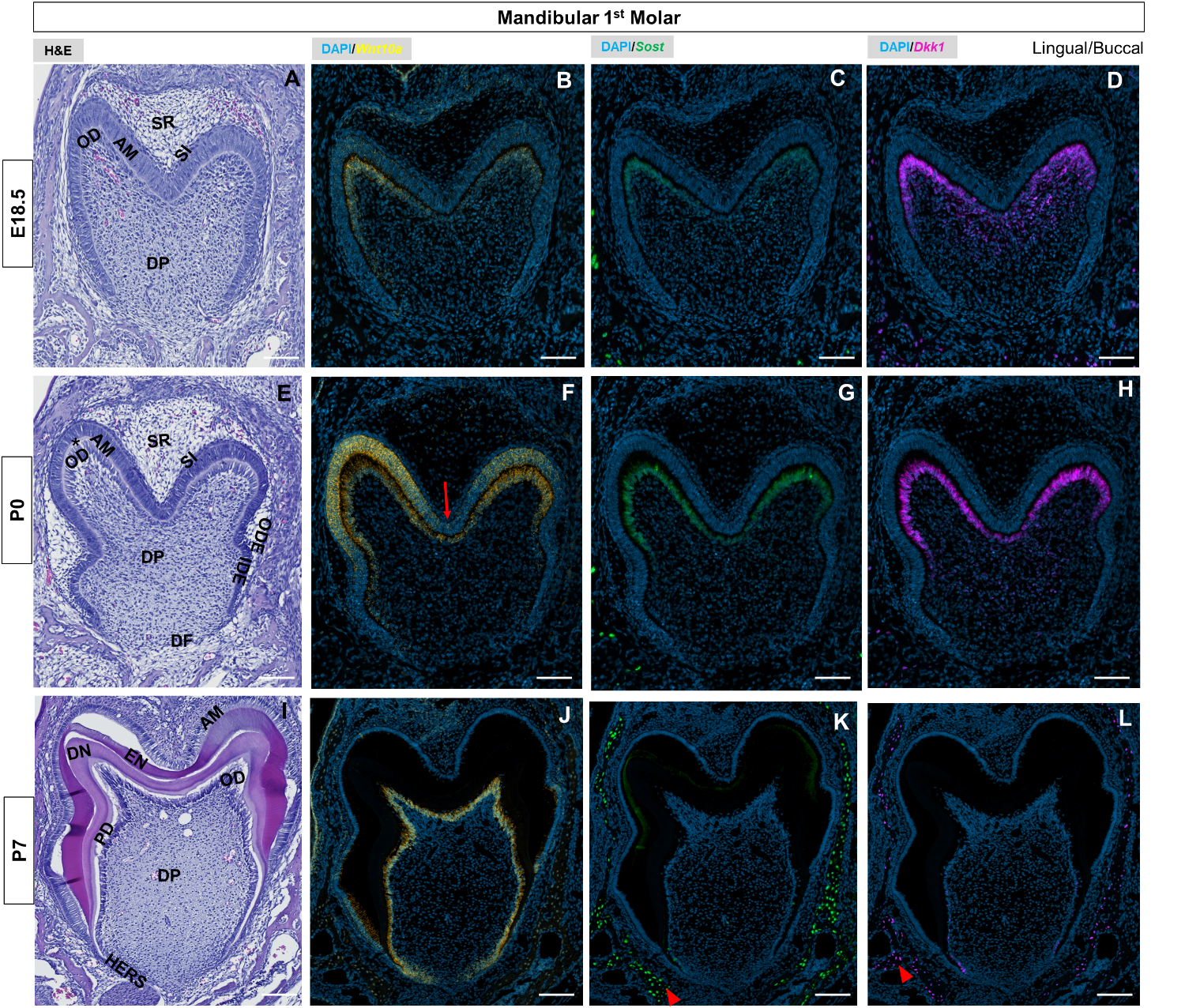
**

**Supplementary Figure 4** Hematoxylin and eosin (HE) staining (**A**, **E, I**). RNAscope *in situ* hybridization of *Wnt10a*, *Sost* and *Dkk1* with positive staining in yellow dots, green dots and violet dots respectively, on coronal sections of the mandibular molar tooth organ at E18.5, P0, P7. Red arrowhead in K and L shows strong signals of *Sost* and *Dkk1* within the osteocytes of developing bone. Weaker *Wnt10a* expression in the ameloblasts located at the occlusal groove of mandibular molars in shown by red arrow in F. Predentin deposition in mandibular molars is indicated by black asterisk in E. Scale bar, 100 µm.OD-Odontoblast, AM-Ameloblast, SR-Stellate reticulum, SI-Stellate intermedium, IDE-Inner dental epithelium, ODE- Outer dental epithelium, DP-Dental papilla, DF-Dental follicle, EN-Enamel, DN-Dentin, PD-Pre-dentin, HERS-Hertwig’s epithelial root sheath.

**A**

Maxillary 1^st^ Molar


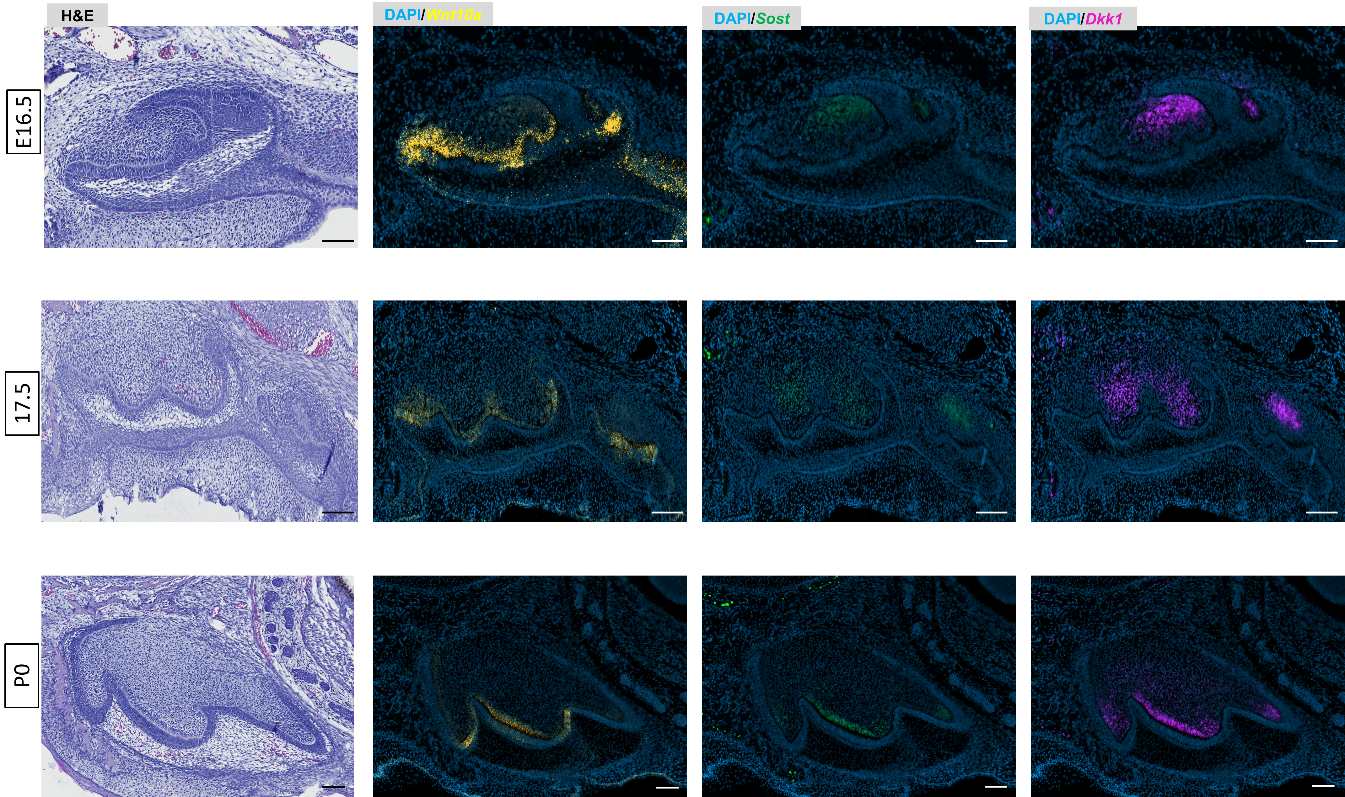


Mandibular 1^st^ Molar

**B**

**
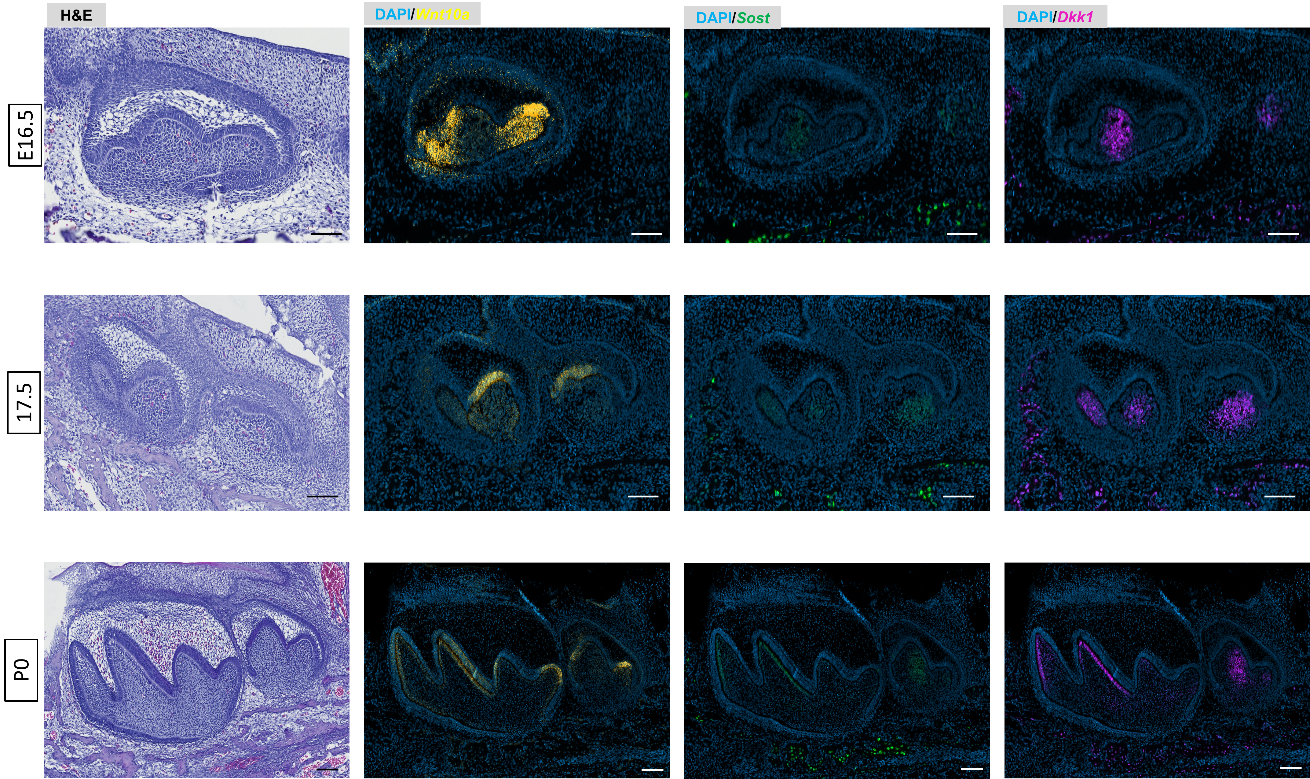
** **Supplementary Figure 5** H&E staining and multiplex in situ hybridization (RNAscope) of maxillary and mandibular molars tooth organ in sagittal reference. Scale bar, 100µm.

**
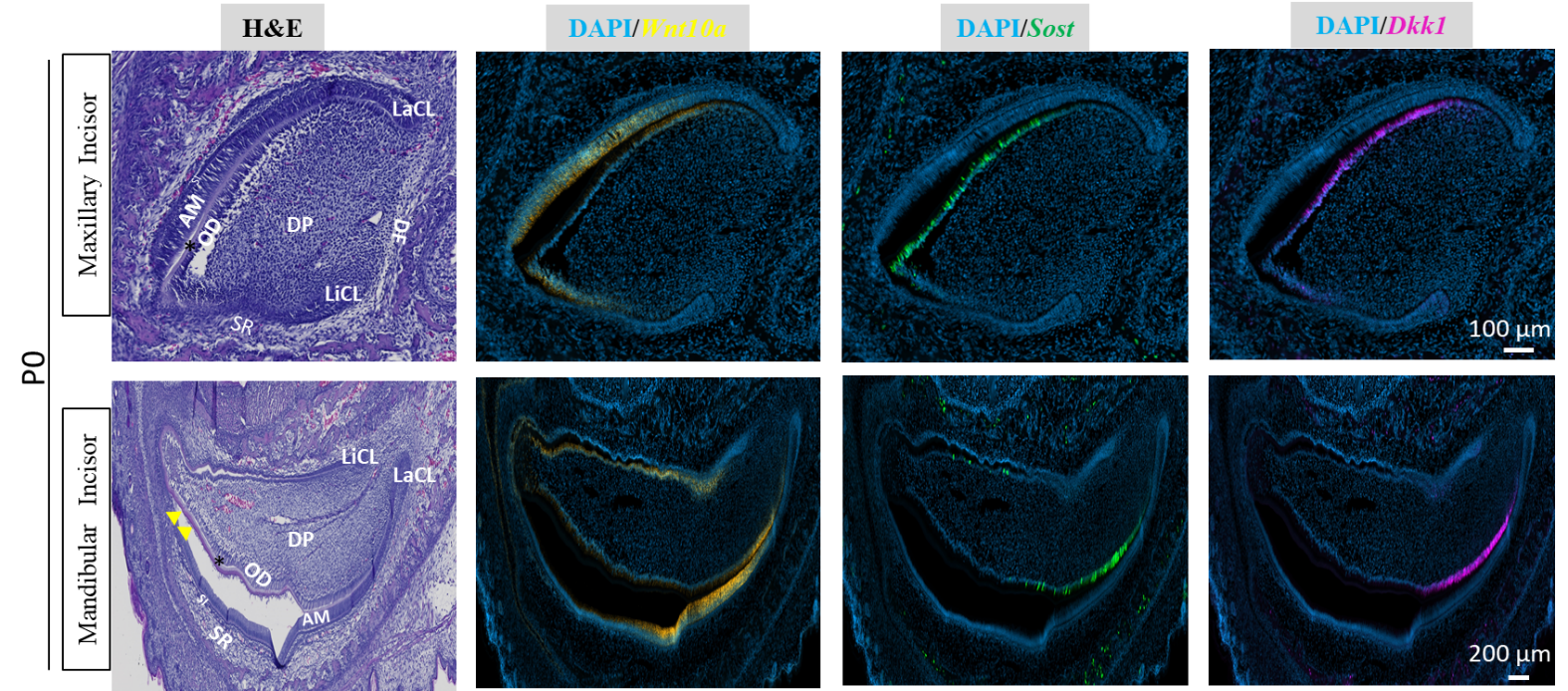
**

**Supplementary Figure 6** Maxillary and mandibular incisor tooth organs of wild type mice at P0 were analyzed using hematoxylin and eosin (HE) staining and RNAscope *in situ* hybridization of *Wnt10a*, *Sost* and *Dkk1* with positive staining in yellow dots, green dots and violet dots respectively, on sagittal sections. Yellow arrowhead shows enamel formation on the labial surface. Predentin deposition is indicated by black asterisk. EPI-Epithelium, MES-Mesenchyme, OD-Odontoblast, AM-Ameloblast, SR-Stellate reticulum, SI-Stellate Intermedium, IEE-Inner enamel epithelium, OEE- Outer enamel epithelium, DP-Dental papilla.


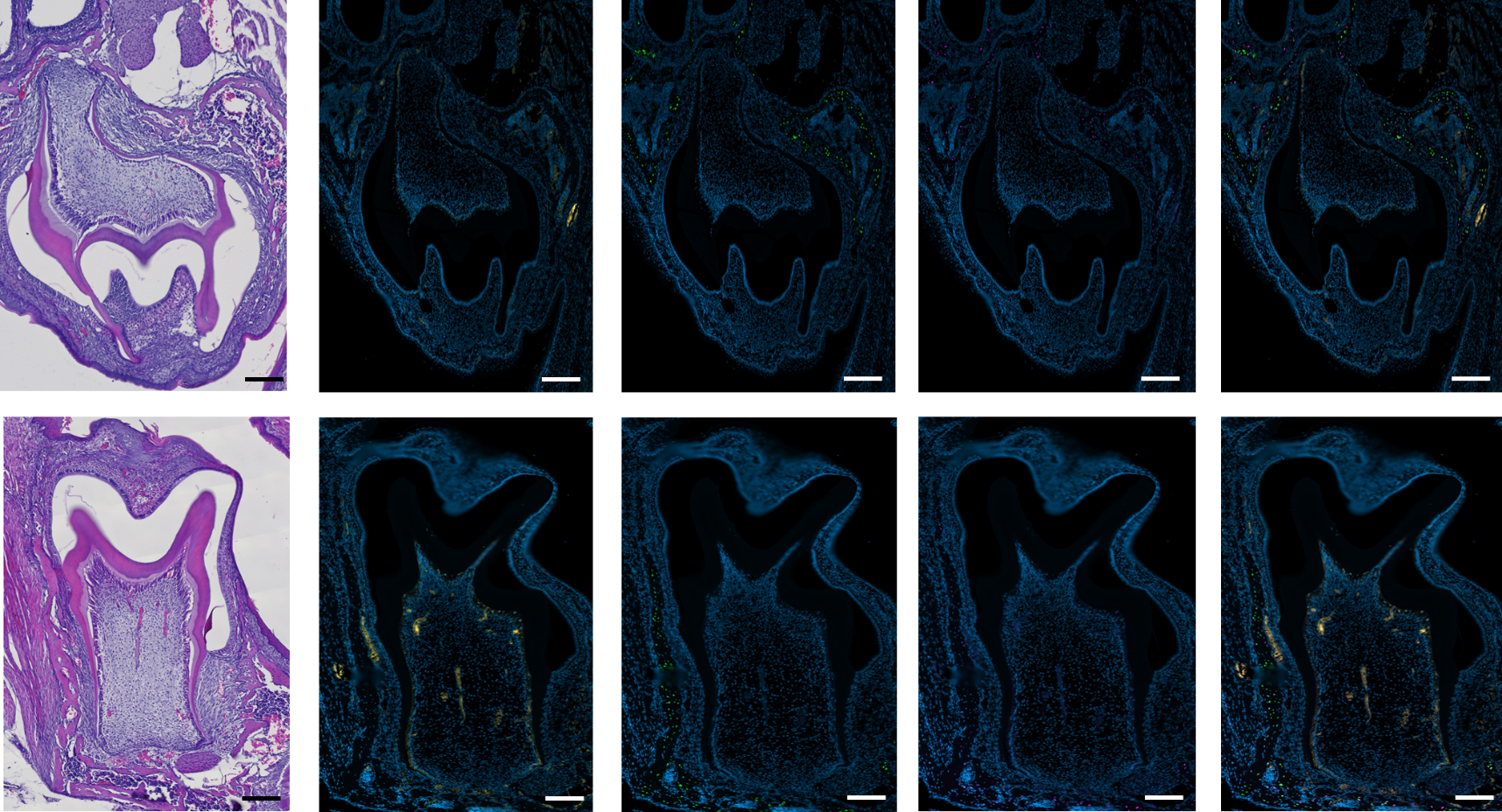


**DAPI/*Dkk1***

**DAPI/*Sost***

**DAPI/*Wnt10a***

**H&E**

P14

Maxillary 1^st^ Molar

Mandibular 1^st^ Molar

**DAPI/*Dkk1***

**Supplementary Figure 7** H&E staining and multiplex in situ hybridization (RNAscope) of

P14 maxillary and mandibular 1^st^ molars tooth organ in coronal reference. Scale bar, 100µm.


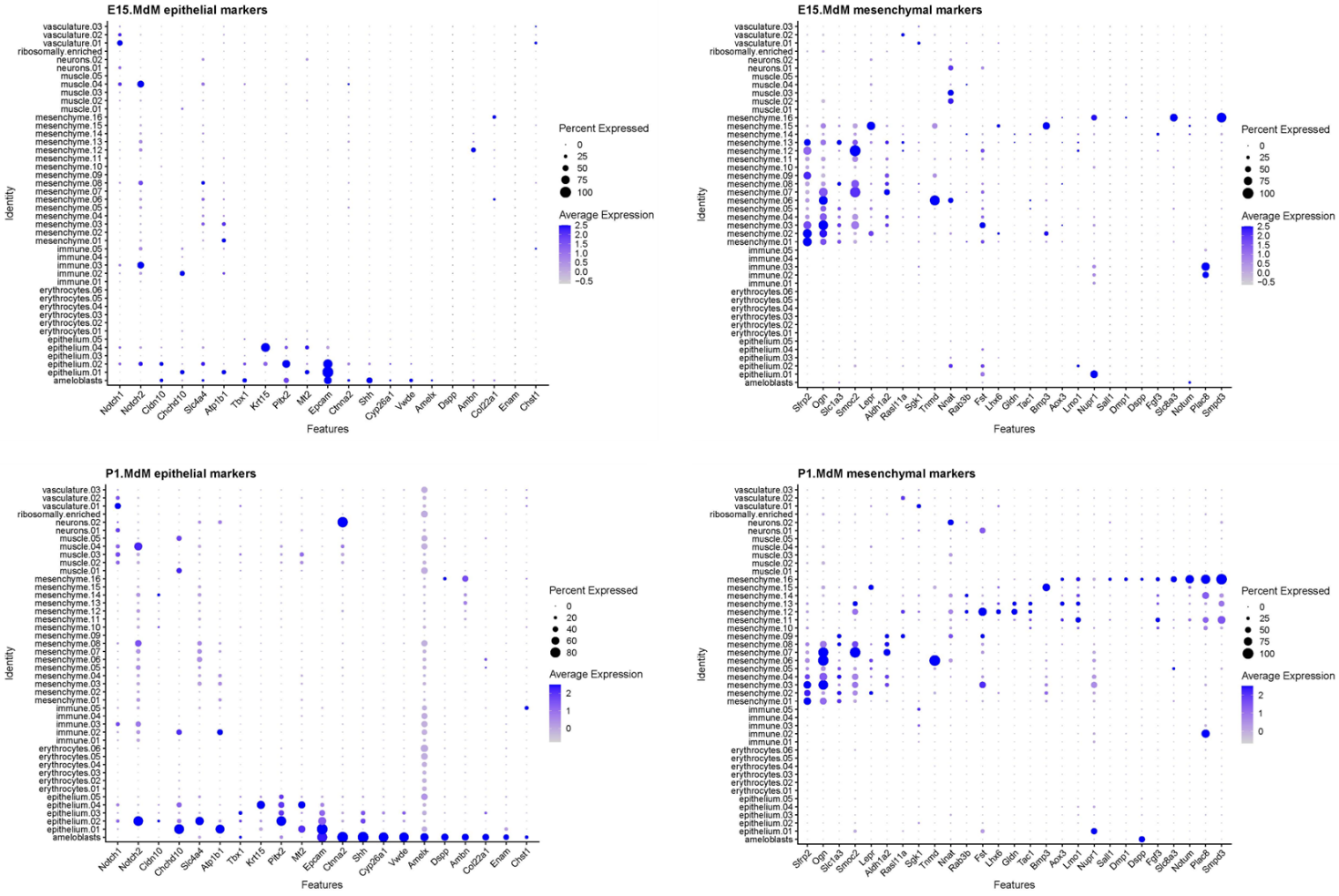


**Supplementary Figure 8** Dot plots of gene markers used for identifying different cell populations in single-cell RNA-sequencing of whole mandibular molar tooth organs.


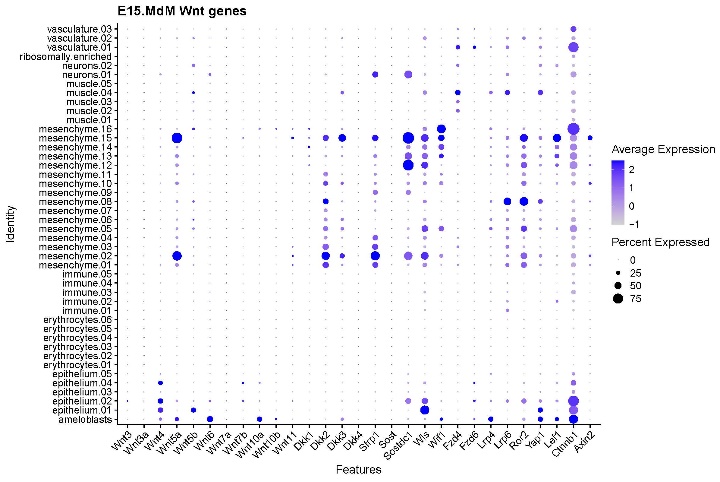

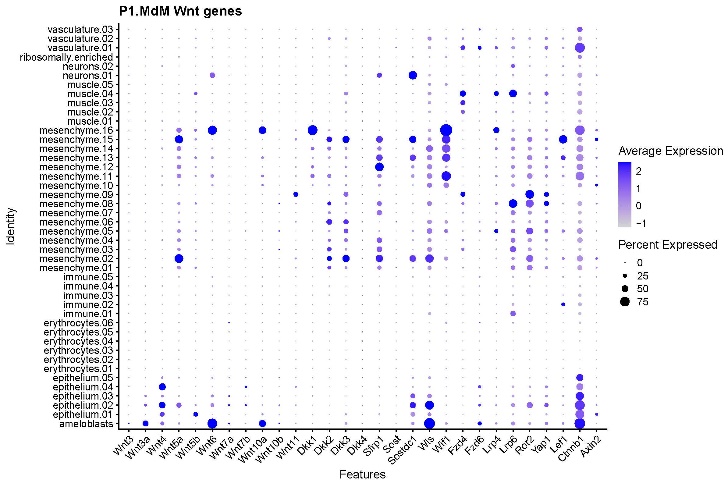


**Supplementary Figure 9** Dot plot showing the expression of selected markers of Wnt signaling genes known to play a role in Mandibular molar tooth development. Mesenchyme 16 cluster contains odontoblasts.
